# Survival of non-Western first generations immigrants with stomach cancer in North East Netherlands

**DOI:** 10.1038/bjc.2011.64

**Published:** 2011-02-22

**Authors:** E J M Siemerink, M A van der Aa, S Siesling, G A P Hospers, N H Mulder

**Affiliations:** 1 University of Groningen and University Medical Centre Groningen, Department of Medical Oncology, P.O. Box 30001, 9700 RB, Groningen, The Netherlands; 2 Comprehensive Cancer Centre North-East, P.O. Box 330, 9700 AH, Groningen, The Netherlands; 3 Health Technology and Services Research, University of Twente, P.O. Box 217, 7500 AE, Enschede, The Netherlands

**Keywords:** stomach cancer, first generation immigrants, outcome, North East Netherlands

## Abstract

**Background:**

Isolated groups, such as first generation non-Western immigrants, are at risk for suboptimal utilisation of the health care system resulting in a worse outcome.

**Methods:**

From 1989 to 2007, all patients with stomach cancer were selected from the Comprehensive Cancer Centre North-East cancer registry. Associations between country of birth and patient, tumour and treatment characteristics were determined using *χ*^2^ analysis. Relative survival analysis was used to estimate relative excess risk of dying according to country of birth (non-Western *vs* Western).

**Results:**

After adjusting for confounding factors (patient, tumour and treatment related), the risk of dying was lower for first generation non-Western immigrants (relative excess risk 0.55, 95% confidence interval 0.43–0.70) compared with Western patients.

**Conclusion:**

Although the better survival of first generation non-Western immigrants with stomach cancer remains unexplained, it argues against accessibility problems within the Dutch health care system.

The Dutch health care system, financially based on an obligatory insurance coverage, is designed to provide essential health care to all citizens. However, isolated groups might be at risk for suboptimal care whether in a social, cultural, religious and/or communicative way (Waller *et al*, 2009). A group that would fit these characteristics are first generation immigrants from non-Western origin because of factors related to the migration process, such as health status, self perceived needs, health seeking behaviour, language barriers, or other cultural or religious differences, which might increase patient delay and influence outcome of care.

Accessibility of health care is particularly relevant in diseases, like stomach cancer, in which care and outcome are highly dependent on early detection; it therefore provides a valuable opportunity of using survival as an endpoint, as delayed diagnosis and under-treatment would influence survival. For this purpose, we analysed data on survival of stomach cancer in the North East Netherlands for first generation non-Western immigrants compared with Western immigrants and native patients.

## Materials and methods

All cases of stomach cancer (ICD-10 code C16) diagnosed between 1 January 1989 and 31 December 2007 (*n*=9239) were selected from the population-based cancer registry of the Comprehensive Cancer Centre North East (CCCNE: 3.2 million inhabitants). First notifications are obtained from the nation wide network and registry of histo- and cytopathology in Netherlands (PALGA) and the national registry of hospital discharge, radiotherapy institutions and haematology departments. Information on patient characteristics and tumour characteristics such as sub-site (International Classification of Diseases for Oncology (ICD-O-3) (Wittekind *et al*, 2005), histology, stage (tumour lymph node metastasis (TNM) classification (Sobin and Wittekind, 2002)), and grade, are obtained routinely from the medical records ∼9 months after diagnosis (Siesling *et al*, 2003). The quality of the data is high, completeness being estimated to be at least 95% (Schouten *et al*, 1993). Follow-up of vital status of all patients was initially obtained from municipal registries and from 1995 from the nationwide municipal population registries network and was calculated as the time from diagnosis to death or to 1 January 2008.

The cancer registry obtains country of birth from the patient files in the hospitals. When a hospital does not collect this information, the country of birth is coded as ‘unknown’. An unknown country of birth therefore depends on the hospital in which the patient is diagnosed and not on the patient. Of the 9239 patients, 1863 had an unknown country of birth and were excluded from the analyses. Of the remaining 7376 patients, 7259 were born in Europe (excluding Turkey), North America, Australia and New Zealand, and were coded as Western immigrants or native patient; those born in other countries were coded as non-Western immigrants (Table 1, *n*=117).

### Statistical analysis

Associations between country of birth, age at diagnosis, stage of disease, localisation of the tumour, histological grade of the tumour and treatment were analysed by *χ*^2^ analysis and calculating 95% confidence intervals (CI). Localisation was divided into proximal (C16.0 and C16.1), distal (C16.2, C16.3, C16.4, C16.5 and C16.6), overlapping lesions (C16.8) and not otherwise specified (C16.9).

As information on cause of death is not available in the cancer registry, relative survival was used as an estimation of disease-specific survival. This adjusts for survival in the general population with the same structure for age and gender, and is calculated as the ratio of the observed rates in cancer patients to the expected rates in the general population (Hakulinen and Abeywickrama, 1985). Multivariable 5-year survival analyses were conducted to discriminate independent risk factors for death, expressed in relative excess risk of dying (RER). Year of diagnosis was divided into categories and put into the multivariable model to see if there were changes in survival over time. Patients with unknown stage of disease were excluded from the survival analyses (*n*=1415). Statistical analysis was performed with Stata version 10 (StataCorp LP, College Station, TX, USA).

## Results

The majority of the 7376 patients were male (66%). The mean age at diagnosis was 71 years (range 18–99 years). Most patients were diagnosed in stage IV (35%). The proportion of patients from non-Western origin did not change over time (data not shown). Table 2 shows the distribution of individual patient and tumour characteristics per group. Non-Western immigrants were diagnosed on average at a younger age and presented more often with well-differentiated tumours than Western immigrants or native patients. No differences were found in localisation, stage or treatment of the tumours.

Table 3 shows the univariate 5-year relative survival, as well as the multivariable 5-year relative survival related to age, gender, histological grade, localisation, stage and treatment. Univariately, the relative 5-year survival for patients from Western origin was 17 and 31% from non-Western origin. As year of diagnosis did not change the multivariable model, year of diagnosis was not put into the final model. In general, risk of dying is slightly decreased in the multivariable analysis compared with the univariate analysis, indicating that the risks are influenced by the other confounding factors in the table. The risk of dying is decreased independently for patients from non-Western origin: RER 0.55, 95% CI 0.43–0.70. Furthermore, patients diagnosed with stage II, III or IV show an increased risk of dying compared with patients with tumours diagnosed in stage I.

## Discussion

Isolated populations are at risk for experiencing access barriers to the health care system, which can delay diagnosis and worsen outcome. Although survival rates of stomach cancer have steadily improved over recent decades, it remains a deadly disease without early diagnosis and treatment, requiring advanced medical measures (Hartgrink *et al*, 2009). In the current study, stomach cancer survival among non-Western immigrants did not suggest that such barriers existed for them. This observation was contrary to our expectations, as this group is often considered at risk for isolation because of factors like a limited social network, religious convictions, language barriers and a lower socioeconomic status. (Fontana *et al*, 1998; Brewster *et al*, 2000; Nagel *et al*, 2007; Collins, Villagran and Sparks, 2008; Butow *et al*, 2010; Kagawa-Singer *et al*, 2010). Surprisingly, we observed a better outcome of patients with stomach cancer from non-Western origin (RER 0.55, 95% CI 0.43–0.70) compared with those of Western origin.

Differences in outcome of stomach cancer can be explained by differences in patient and tumour characteristics besides the functioning of the health care system. Although non-Western immigrants were substantially younger when diagnosed, we did not observe differences in tumour characteristics, which could explain this. Patients from non-Western origin, especially first generation immigrants, however, are more often born in developing countries where the prevalence of *Helicobacter pylori* is higher and acquired at a younger age, than in developed countries. Although this may explain part of the earlier age of diagnosis, as *H. pylori* is considered a class 1 carcinogen, it cannot be the sole explanation, as some populations in which *H. pylori* is prevalent have high incidences of gastric cancer, while other highly infected populations do not (Singh and Ghoshal, 2006; Yamaoka *et al*, 2008). Although one would expect young age to be a favourable factor for survival, multivariate analysis adjusting for age continued to show a beneficial outcome of first generation non-Western immigrants.

Perhaps some favourable factors particularly applicable to the Dutch situation, could have facilitated utilisation of the medical services notwithstanding different degrees of isolation. We suggest that the high degree of employment of first generation non-Western immigrants and the Dutch ‘arbodienst’, a free health service for employees within every company, could have facilitated access to medical care despite the language barrier. Further research is needed, since the observed survival advantage is unexpected and remains unexplained, especially as there is no indication that any form of treatment or intervention is applied more often in this group.

In conclusion, the better survival of first generation non-western immigrants after stomach cancer compared with the control population remains unexplained, but suggests that access to the health care system in Netherlands was not hampered for these immigrants.

## Figures and Tables

**Table 1:** Distribution of origin of non-Western patients with stomach cancer

**Country of birth**	**Number of patients**
Africa	7
South America	2
Middle East	58
Surinam/Netherlands Antilles and Aruba	18
Far East	26
Russia	6
Total	117

**Table 2:** Comparison of characteristics of Western immigrants and autochthonic patients with non-Western patients with stomach cancer in North East Netherlands

	**Western immigrants and autochthonic patients**		**Non-Western immigrants**		
	**%**	**95% CI**	**%**	**95% CI**	***P*-Value**
*Gender*					
Male	66	65–67	62	53–70	0.314
Female	34	33–35	38	30–47	
					
*Age*					
0–14	—	—	—	—	<0.001
15–29	0.1	0.1–0.2	4.3	0.6–7.9	
30–44	2.5	2.1–2.8	21	13–28	
45–59	15	14–16	17	19–35	
60–74	40	39–41	35	26–44	
75+	43	42–44	13	7–19	
					
*Tumour localisation*					
Proximal	29	28–30	21	14–29	0.402
Distal	44	43–45	50	41–59	
Overlapping lesion	20	19–21	22	15–30	
Not otherwise specified	7	7–8	7	2–11	
					
*Histological grade*					
Poor	2.8	2.5–3.2	1.7	0.1–4.1	0.034
Medium	20	19–20	15	9–22	
Well	41	40–42	46	37–55	
Undifferentiated	0.9	0.7–1.1	3.4	0.1–6.7	
Unknown	36	35–37	33	25–42	
					
*Stage* a					
1	17	16–18	15	8–21	0.125
2	13	13–14	12	6–18	
3	16	15–17	20	13–27	
4	35	34–36	42	33–51	
Unknown	19	18–20	12	6–18	
					
*Treatment*					
Resection	43	42–45	50	41–60	0.207
Resection+chemotherapy	1.0	0.8–1.3	1.7	0.01–4.1	
Chemotherapy	5	4.4–5.3	7	2–11	
None/unknown	37	36–38	31	22–39	
Other	14	13–15	10	5–16	

Abbreviation: CI=Confidence interval.

a Stage 1: T1N0M0, T1N1M0, T2a/bN0M0; Stage 2: T1N2M0, T2a/bN1M0, T3N0M0; Stage 3: T2a/bN2M0, T3N1M0, T4N0M0, T3N2M0; Stage 4: T4N1,2,3M0, T1,2,3N3M0, anyTanyNM1.

**Table 3:** Univariate and multivariate relative 5-year survival analyses for Western *vs* non-Western patients with stomach cancer in the Netherlands, period 1989–2007

	**Univariate**		**Multivariate**	
	**RER^a^**	**95% CI**	**RER^a^**	**95% CI**
*Country of birth*				
Western	1	Reference	1	Reference
Non-Western	0.68	0.54–0.87	0.55	0.43–0.70
				
Age	1.00	0.99–1.00	1.00	0.99–1.00
				
*Gender*				
Men	1	Reference	1	Reference
Women	0.98	0.92–1.04	0.94	0.88–1.00
				
*Localisation*				
Proximal	1	Reference	1	Reference
Distal	0.74	0.69–0.80	1.00	0.93–1.08
Overlapping lesion	1.26	1.16–1.37	1.23	1.13–1.34
Not otherwise specified	1.35	1.19–1.53	1.24	1.09–1.41
				
*Histological grade*				
Well	1	Reference	1	Reference
Medium	1.38	1.12–1.72	1.00	0.81–1.23
Poor	1.93	1.57–2.37	1.21	0.98–1.48
Undifferentiated	1.77	1.23–2.56	1.06	0.73–1.52
Unknown	2.42	1.96–2.98	1.03	0.83–1.27
				
*Stage* b				
I	1	Reference	1	Reference
II	2.21	1.95–2.52	2.20	1.94–2.49
III	3.55	3.14–4.00	3.12	2.77–3.51
IV	9.05	8.09–10.11	4.93	4.38–5.54
				
*Therapy*				
Resection	1	Reference	1	Reference
Resection+chemotherapy	1.58	1.22–2.03	1.29	0.99–1.67
Chemotherapy	2.3.09	2.73–3.48	1.61	1.40–1.84
None/unknown	6.28	5.84–6.75	3.68	3.37–4.03
Other	4.26	3.90–4.66	2.75	2.48–3.05

Abbreviations: CI=Confidence interval; RER=relative excess risk of dying.

a Adjusted for age, gender, localisation, histological grade, stage and treatment.

b Stage 1: T1N0M0, T1N1M0, T2a/bN0M0; Stage 2: T1N2M0, T2a/bN1M0, T3N0M0; Stage 3: T2a/bN2M0, T3N1M0, T4N0M0, T3N2M0; Stage 4: T4N1,2,3M0, T1,2,3N3M0, anyTanyNM1.
